# Implementation of a campus-based and peer-delivered HIV self-testing intervention to improve the uptake of HIV testing services among university students in Zimbabwe: the SAYS initiative

**DOI:** 10.1186/s12913-022-07622-1

**Published:** 2022-02-18

**Authors:** Fadzai Mukora-Mutseyekwa, Paddington Tinashe Mundagowa, Rugare Abigail Kangwende, Tsitsi Murapa, Mandla Tirivavi, Waraidzo Mukuwapasi, Samantha Nokuthula Tozivepi, Charles Uzande, Queen Mutibura, Elizabeth Marian Chadambuka, Mazvita Machinga

**Affiliations:** 1 Clinical Research Centre, Africa University, 132 H. Chitepo Street, Mutare, Zimbabwe; 2 Monitoring & Evaluation Unit, Ministry of Health & Child Care, Harare, Zimbabwe; 3 Department of Students’ Affairs, Africa University, P. O. Box, 1320 Mutare, Zimbabwe; 4 HIV/STI/TB Unit, Ministry of Health and Child Care, Manicaland Province, Mutare, Zimbabwe; 5 Department of Public Health & Nursing Sciences, Africa University, 1 Fairview Road, Old Mutare, Zimbabwe; 6 Chair of Pastoral Care & Counseling Services, Number 3, 13th Avenue, Mutare, Zimbabwe

**Keywords:** HIV, Self-testing, Adolescent, Young adult, EPIS framework, Implementation science

## Abstract

**Background:**

The uptake of HIV testing services among adolescents and young adults in Zimbabwe is low due to stigma associated with the risk of mental and social harm. The WHO recommends HIV self-testing (HIVST) as an innovative approach to improve access to HIV testing for this hard-to-reach populations. This study describes the development and implementation of a coordinated multifaceted and multidisciplinary campus-based approach to improve the uptake of HIV testing among university students in Zimbabwe.

**Methods:**

We utilized both quantitative and qualitative methods guided by the Exploration, Preparation, Implementation, and Sustainment Framework. A formative survey, in-depth interviews, and a scoping review were conducted as part of the situation analysis. Implementers (peer educators and health workers) were trained and community dialogue sessions were conducted to ascertain the determinants (enablers and barriers) influencing both the inner and outer contexts. Self-test kits were disbursed over 6 months before a summative evaluation survey was conducted. Qualitative data were analyzed thematically while the chi-squared test was used to analyze quantitative data.

**Results:**

The formative evaluation showed that 66% of students intended to test and 44% of the enrolled students collected HIVST test kits. Giving comprehensive and tailored information about the intervention was imperative to dispel the initial skepticism among students. Youth-friendly and language-specific packaging of program materials accommodated the students. Despite the high acceptability of the HIVST intervention, post-test services were poorly utilized due to the small and isolated nature of the university community. Implementers recommended that the students seek post-test services off-site to ensure that those with reactive results are linked to treatment and care.

**Conclusions:**

Peer-delivered HIVST using trained personnel was acceptable among adolescents and young adults offered the intervention at a campus setting. HIVST could increase the uptake of HIV testing for this population given the stigma associated with facility-based HTS and the need for routine HIV testing for this age group who may not otherwise test. An off-site post-test counseling option is likely to improve the implementation of a campus-based HIVST and close the linkage to treatment and care gap.

**Supplementary Information:**

The online version contains supplementary material available at 10.1186/s12913-022-07622-1.

## Background

Globally, the HIV/AIDS pandemic has caused ineffable suffering to people of all age groups and young people have not been spared due to their disproportionate vulnerability to HIV acquisition, both socially and economically [1–4]. HIV-related deaths among young people have more than tripled since 2000, and HIV/AIDS is ranked the second cause of death among adolescents worldwide [5].

A national survey conducted in Zimbabwe in 2015 revealed that only 46% of young women and 47% of young men had comprehensive knowledge of HIV Testing Services (HTS) [6]. 39% of male adolescents and young adults and 63% of female adolescents and young adults who had had sexual intercourse within 12 months preceding the survey reported having been tested for HIV. HIV/AIDS-related stigma is associated with a reduced likelihood of HIV testing because the diagnosis is linked with the risk of social harm [7–11]. Other barriers influencing the uptake of HTS are user fees, lack of confidentiality, long waiting times, negative health worker attitudes, low-risk perception, and distance from a health facility [12, 13].

Innovative and client-centered HIV-testing approaches like *self-testing* could be useful to address most of these barriers [14–16]. HIV self-testing (HIVST) refers to a process in which a person collects his or her own specimen (oral fluid or blood) and then performs an HIV test and interprets the result, often in a private setting, either alone or with someone he or she trusts [17]. The Sub-Saharan Africa Region adopted HIVST in 2015 and by 2017, 15 counties in the region had shifted their national policies in support of the approach [18]. The rationale of the HIVST approach is accessing and scaling up HTS among population groups that are hard to reach, do not want to test, do not test regularly, or are unable to attend routine test services [19, 20]. The concept of HIVST was well received by most key stakeholders involved in HIV programming and they emphasized the need for adequate research before adoption [21]. However, very little is known on the essential components required for selecting and guiding the strategy to address context-specific barriers.

Preliminary positive findings from pilot research in Zimbabwe informed the adoption of HIVST by the Ministry of Health and Child Care (MoHCC) [22]. The pilot study recommended for regulation of HIVST strategies because test results can have significant implications on mental health, health-seeking, sexual behavior, and HIV transmission. Areas of supervision include adherence to quality standards of the self-test kits, logistics of kit distribution, and the nature of HIVST marketing/advertising messages [23]. Traditionally, top-down approaches used in the development of public health interventions that were derived from behavior change theories are assumed to be one size fits all [24]. However, this approach has caused implementation and sustainability challenges as well as poor end-user adherence [25, 26]. The MoHCC recommended for HIVST along the lines of differentiated service delivery to diminish the number of people oblivious of their HIV status [27]. This study set out to articulate the development and implementation of a systematic and multifaceted HIVST intervention dubbed the Swipe And Know Your Status (SAYS) and test the intervention’s acceptability and feasibility to promote the uptake of HTS among university students.

## Methods

**Fig. 1:**
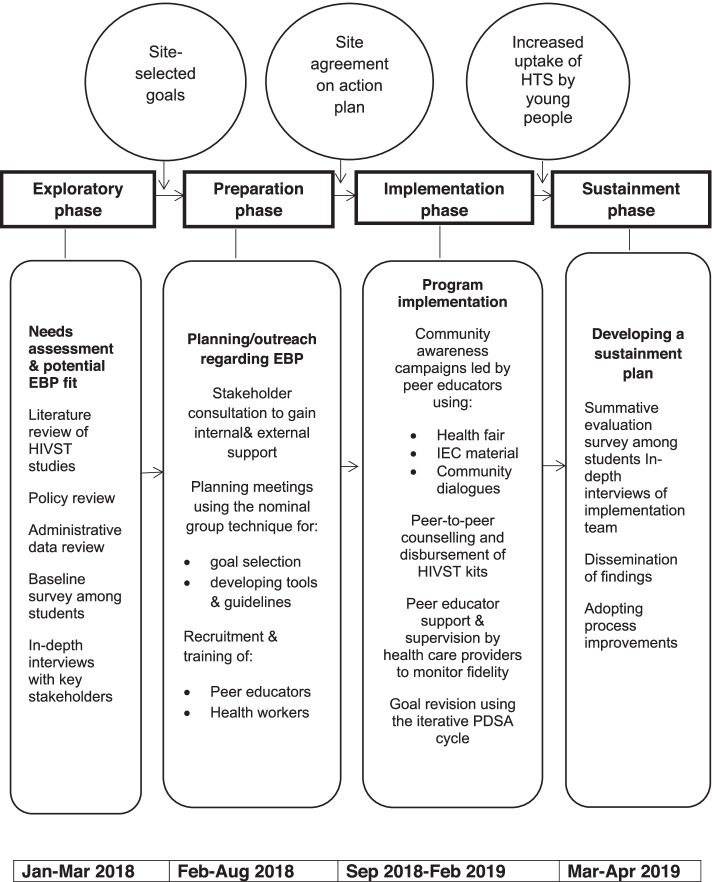
The phases of the EPIS Framework as utilized in the study. Adapted from Aarons et al., 2011 [28]. EBPs: Evidence-based practices; HIVST: HIV Self-testing; IEC: Information, Education and Communication, PDSA: Plan-Do-Study-Act

This study employed the Exploration, Preparation, Implementation, and Sustainment (EPIS) Framework [28]. The framework was specifically selected by the research team because the four phases interact dynamically with the inner (intra-organizational and individual adopter characteristics) and outer (service-, inter-organizational-, and advocacy environments) contexts that influence the success or failure of the implementation process. Figure 1 shows how the EPIS Framework was used in the study. The researchers operationalized the framework by beginning with sustainment in mind and adapting outer and inner contexts, strategies as well as evidence-based practices of the implementation process.

### Setting

The study was conducted at Africa University, a tertiary education institution 18 km north of the eastern border city of Mutare, Zimbabwe. The target population was 1690 university students from 29 African countries and enrolment statistics at the time showed that 53% of the students were females, and 32% were international students. About 65% of the students lived on campus and 25% were housed in University-recommended hostels in Mutare City. The university is a small and geographically isolated community outside town and away from other health facilities and it had one clinic which was manned by four registered nurses who were trained to offer HTS.

### Study design and sample

This was an exploratory sequential mixed method study and data were collected in three phases. The first phase was divided into two steps namely the exploration and preparation steps while the second phase involved the implementation and the final phase was the sustainment. Findings from the first phase guided implementation strategy selection. Survey tools used in both phase 1 (formative evaluation) and phase 3 (summative evaluation) were developed from previously used and published literature that assessed the acceptability and feasibility of HIVST. The data extraction tools were pre-tested at a technical college in Mutare City and its corresponding health facility for clarification, reliability, and validity. Before pretesting, the survey questionnaires were reviewed for face validity by the HTS experts from the MoHCC, and two local non-governmental organizations (NGOs) offering HTS to adolescents and young adults. This was done to ensure that the statements were clearly understood by participants and the questions were appropriate for the age group. To achieve effective translation of data extraction tools and add methodological rigor, forward and backward translation was done using four blinded multilingual experts from Africa University.

All enrolled university students were eligible to participate in both the formative and summative evaluation surveys and the age range of the participants was 18 to 34 years. Students who were on attachment away from campus and those who were aware of their HIV-positive status were excluded from the study.

Ethical clearance was issued by the Medical Research Council of Zimbabwe and the Africa University Research Ethics Committee. Permission to carry out the study was granted by the Provincial Medical Director of Manicaland and the Africa University administration. Written informed consent was obtained from all study participants. Due to the sensitive nature and stigma associated with HIV/AIDS, a distress protocol was developed to manage possible adverse emotional reactions especially in cases of unexpected reactive post-test results. The participants were informed about the protocol and were encouraged to seek in-person or anonymous support from the counseling team led by MM via a 24-h helpline service.

### Phase 1

#### Step 1: Exploration (January to March 2018)

A campus-wide formative evaluation survey that employed quantitative methods was conducted to assess the implementation readiness of the inner context and innovation factors (e.g. prospective implementation barriers and facilitators) using a structured, pretested interviewer-administered questionnaire with 17 open- and closed-ended questions. The secondary aim was to ascertain the program implementation determinant (i.e. barriers and enables) as well as acceptability and feasibility pre-implementation as a benchmark against which the results of the summative evaluation were later compared with. The survey was conducted from 5 February to 9 March 2018.

Structured in-depth interviews were also used among purposively selected eight key informants from NGO representatives, government HTS focal persons at district and provincial levels, and university administrators to ascertain the outer context factors (e.g. organizational culture, climate, and work attitudes) that would influence the implementation of the intervention. The MoHCC and local HIV/AIDS-related NGOs were consulted since they were the custodian and agencies of the national HTS program, respectively.

The research team did a rapid scoping of literature underpinning the area of HIVST to ascertain strategies used by other researchers, identify knowledge gaps, challenges encountered, and the suggested solutions from the lessons learned as well as clarification of concepts. HIVST-related article search was done in electronic databases (e.g. MEDLINE, EMBASE, PubMed, and Scopus), reference lists, hand-searched journals, and existing networks using the framework proposed by Arksey and O’Marley (2005) [29] with the assistance of the institutional librarian.

#### Step 2: Preparation (February to August 2018)

During the exploration phase, a multidisciplinary team comprising of FMM (Public Health Physician), MT (Research Administrator), TM (Nurse Manager), and EC (Public Health Nurse) converged twice during the month of February 2018 for brainstorming and program planning meetings. For the duration of the meetings, the team assessed the outer context (e.g. national policies on HIV testing, possible inter-organizational networks) and the inner context (e.g. the absorptive capacity at AU, characteristics of target population). They also discussed the key program elements, such as (1) the stakeholders to be consulted, (2) materials to be procured, (3) strategies to be implemented, and (4) outlined individual responsibilities.

**Fig. 2:**
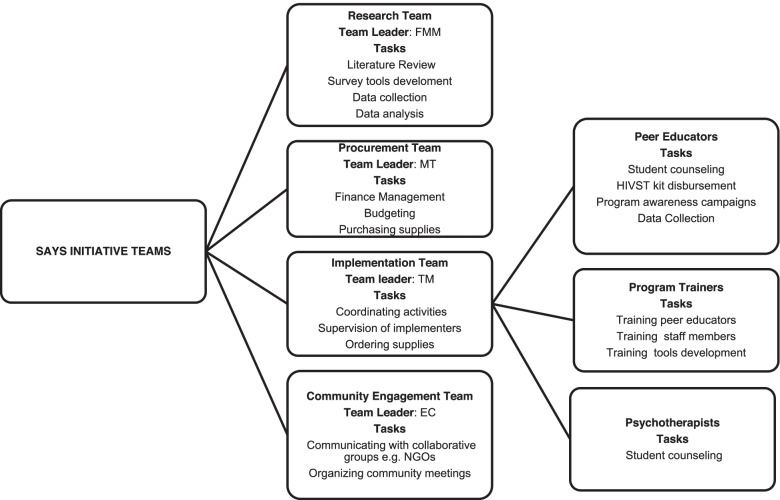
The four teams that participated in the implementation of the SAYS Initiative

CU and QM (MoHCC Provincial HIV/STI/TB Focal persons) were contracted as program trainers who developed the HIVST Project Training of Trainers Facilitators Manual Hand Book (Additional file 1) and Training Evaluation Forms for participants (Additional file 2). FMM, MT, TM, and EC led the research team, procurement team, implementing team, and community engagement team, respectively. Figure 2 shows how the four teams collaborated towards the success of the SAYS Initiative.

The team leaders met for implementation strategy selection by way of interpreting the findings of the formative evaluation survey, selecting goals, develop tools and guidelines using the nominal group technique. The implementing team organized two community dialogue sessions which were held on campus and the audio recorded sessions were attended by 83 participants who were purposively selected according to their roles on campus and in HTS programming. These sessions aimed to introduce the SAYS Initiative and identify potential barriers and enablers of implementing the intervention. Volunteers from the university’s Peer Educators Club were selected to become the SAYS Initiative Champions.

As a way of capacity building, improving provider knowledge, and understanding organizational context barriers, 13 (2 males and 11 females) purposively selected staff members with backgrounds in medical, nursing, and laboratory sciences underwent a 5-day training in May 2018. A second training session was conducted for the three health providers from the university clinic, 26 Peer Educators (11 were females). The Peer Educators were students who were later responsible for providing pre-and post-testing psychosocial and emotional support to other students within the institutional environment. However, it was emphasized that complex cases would be referred to official university student counselors and the psychotherapists who were part of the implementation team. The Peer Educators were equipped with information on self-awareness and its role in counseling as participants were mentored on counseling principles, techniques, and processes through presentations, group discussions, and peer counseling role-plays To cater to the culturally and linguistically diverse pan-African environment at the university, the Peer Educators were from different nationalities and while they were all fluent in English (the language of instruction at the institution), a third of them were also fluent in either French or Portuguese languages.

The workshops also adopted and adapted validated and piloted HIVST materials [22] to develop program messages and materials that were to be printed on flyers and banners. The resulting information, education, and communication (IEC) materials were submitted to the Provincial Health Education and Promotion Office for appraisal before printing.

The implementing team engaged the community by scheduling a meeting with 17 local adolescents and young adults (16 to 27 years) who were not university students. The group was made up of Community Adolescent Treatment Support (CATS) members (68% of them were living with HIV), five hospital nurses working in the Opportunistic Infection Clinics in Mutasa District, six members of the Africa University Peer Network Club, and their patron (TM). The aim was to discuss pertinent issues related to HIV/AIDS screening/testing methods and experiences from young people already diagnosed and living with HIV.

### Phase 2

#### Implementation (September 2018 – February 2019)

With the guidance of TM, the Peer Educators broadcasted the HIVST IEC materials. The HIVST video was shared via university clubs’ WhatsApp groups and the university Facebook page. Emails were sent out to students through the Registrar’s desk to advertise the program and Peer Educators received program t-shirts and hats to promote visibility. By way of consensus, the Peer Educators drafted a roaster and visited busy spots like the university dining hall, sporting and games arena, and hostels during lunch hours and weekends. All students were invited to an evening health fair that was held in August 2018 to launch the SAYS Initiative.

##### Intervention

The SAYS Initiative was a new intervention at the university and the name was derived from how the intervention was delivered. Upon receiving pre-test counseling which lasted for 15–20 min from a Peer Educator or a health provider, the participant was given a free HIV oral test kit (OraSure® Technologies, Bethlehem, PA, USA) and offered the option of self-testing in a private room at the clinic or take the test kit to their hostel/home. The HIVST kit package contained a pamphlet of instructions written in English, Shona, French and Portuguese languages to accommodate the language diversity among students at the university. Images displaying the testing steps were also added to complement the text and enhance understanding. Clearly labeled protected bins were placed in the testing rooms and strategic points at the hostels for disposal. While facilitating infection prevention and control, this effort also provided estimated insights into the use of kits as the bins were collected for physical counts of the used kits. The intervention was implemented over 6 months from 2 September 2018 to 28 February 2019.

### Kit disbursement and supervision

HIVST kits were kept at the university laboratory for quality assurance and providers collected the kits based on demand. The MoHCC HIV testing and counseling manuals were adopted for the pre-and post-test counseling. Kit Disbursement Registers were used to anonymously capture socio-demographic data and mobile contact of participants.. Follow-up phone text messages were sent by the nurses to participants within 24 to 48 h of collecting the kit to check how the student was coping and enquire if they required any post-test services.

The university clinic was the central point of HIVST kit distribution. This was done to minimize ‘double-dipping’, ensure health provider support and supervision as well as fidelity monitoring. During the kit disbursement period, Peer Educators developed a rotational duty roaster. Peer Educators briefed the nurses on challenges and potential program adaptations during their daily and monthly meetings. Adaptations were integrated into the program by way of consensus.

### Phase 3

#### Sustainment (March – April 2019)

A structured interviewer-administered questionnaire composed of 20 questions was used for the summative evaluation survey. The study participants were asked about the acceptability, potential concerns, perceived effectiveness of implementation and recommendations for adoption as well as sustainability of the intervention using semi-structured self-administered questionnaires. Accessibility was assessed using self-report measures, which included satisfaction, attitudes, perceptions, as well as experiences both pre-and post-intervention implementation. Evaluation findings were used as reinforcement to augment program sustainability and feed into possible scaling-up of program activities. In-depth interviews of nurses and Peer Educators, as well as record reviews were conducted 6 months post-implementation.

### Data collection and analysis

The research team collected data during the formative evaluation survey with the assistance of Peer Educators. WM (Research Data Manager) and MT conducted in-depth interviews. FMM, PTM (Public Health Officer), SNT (Monitoring and Evaluation Officer), and WM were responsible for data entry and analysis. Audio recorded qualitative data were transcribed verbatim while field notes from dialogue sessions and open-ended survey questions were entered into NVivo 12, and segments were coded before thematic analysis was done by comparing grouped responses that emerged from the data. Quantitative data was imported into Epi Info version 7.2.1.0 (CDC, USA) for bivariate and multivariable logistic regression analysis at 95% confidence interval and 5% level of significance. The dependent variables were the intention to self-test for HIV for the formative evaluation survey and having self-tested for HIV for the summative evaluation survey. For both surveys, the independent variables constituted participants’ socio-demographic characteristics as well as knowledge, attitudes, perceptions, and experiences during the HIVST.

## Results

### Phase 1

#### Formative evaluation survey

Of the 227 participants who participated in the survey, 59% were females and 56% were in their first year of study. Two-thirds (66%) of participants intended to self-test and 56% had had an HIV test before the survey. For those who were not tested, perceived low risk of HIV was the major reason for not testing as reported by 75% of the untested participants. Most students (74%) reported that they would trust the HIVST test result and were aware of how to proceed if they get a reactive result (78%). The majority of students (62%) predicted that the HIVST would be difficult to use and interpret. Table 1 shows the bivariate analysis of the intention to test against the independent variables.

**Table 1:** Bivariate analysis of the baseline survey results

Variable	Characteristic	Intention to test for HIV		COR (95% CI)	***P***-value
		No	Yes		
Age	< 20	25	53	0.9 (0.5–1.6)	0.7
	> 20	52	97		
Sex	Male	29	64	0.8 (0.5–1.4)	0.5
	Female	48	86		
Year of study	1st	42	85	0.9 (0.5–1.6)	0.8
	2nd, 3rd & 4th	34	65		
Previous HIV test	No	66	34	20.5 (9.7–43.1)	< 0.01*
	Yes	11	116		
Believed HIVST kits should be available for all	No	19	24	1.7 (0.9–3.4)	0.1**
	Yes	58	126		
Identified major advantage for HIVST	Anonymity & privacy	53	99	1.1 (0.6–2.0)	0.7
	Other advantages	24	51		
The identified major disadvantage of HIVST	The absence of a counselor increases distress	51	80	1.7 (1.0–3.0)	0.06**
	Other disadvantages	26	70		

The results of logistic regression revealed that students who had not tested before were more likely to intend to test when compared to those who had tested before (AOR 2.8; 95% CI: 1.1–4.9; *p* < 0.01). Despite identifying the absence of a counselor during HIVST as a source of distress, participants who noted this disadvantage were more likely to intend to test when compared to their counterparts who cited other disadvantages (AOR: 2.3; 95%CI:1.1–4.9; *p* = 0.02).

### Phase 2

#### Response to the SAYS initiative awareness process

Seven hundred and forty-four HIVST were disbursed over the targeted period of 6 months and 56% of those who collected the kits were female students. The initial response from the students revealed skepticism especially due to the possibilities of misinterpretation of test results. There were questions raised on whether self-testing could put pressure on the students already burdened by the demands of academic work. As narrated by one of the students, “The toughest hurdle in self-testing is misreading of negative results as positive. What if someone wrongly confirms their suspicion and end up depressed, self-harm or worse committing suicide?” The prospects of the subsequent negative mental health issues especially after a reactive test made the students doubt the acceptability of self-testing. Following interactions with Peer Educators and health care workers, most students’ fears were debunked and the desire to self-test became increasingly evident. The proportion of students approaching the clinic to enquire about the test kits before the scheduled day of disbursement grew exponentially.

**Fig. 3:**
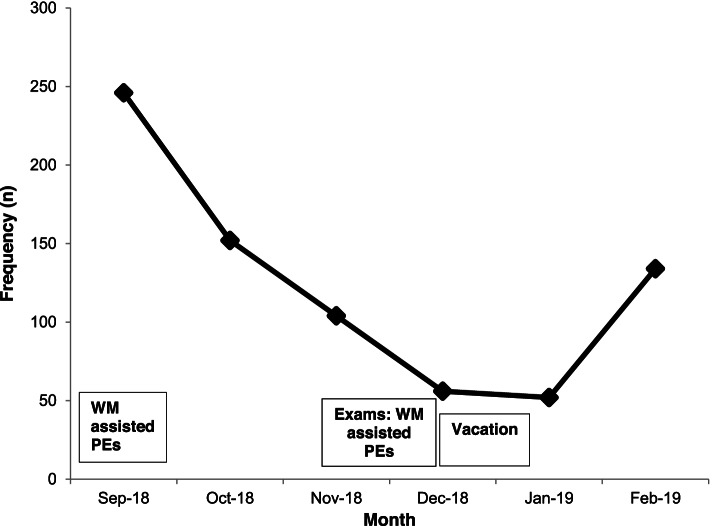
HIVST Kit collection for the period of the kit disbursement

The awareness campaign was well accepted as one student recalled, “The program advertisers were very welcoming and impartial and we were so inquisitive to what this SAYS was all about!” In-depth interviews with the implementers predicted a possible language barrier, especially among the first-year students from French and Portuguese-speaking countries who were still attending intensive English classes. Figure 3 shows the trend of HIV Kit collection over the 6 months of kit disbursement indicating the periods when WM had to assist with kit disbursement at the university clinic.

### Uptake of HIVST kits

**Fig. 4:**
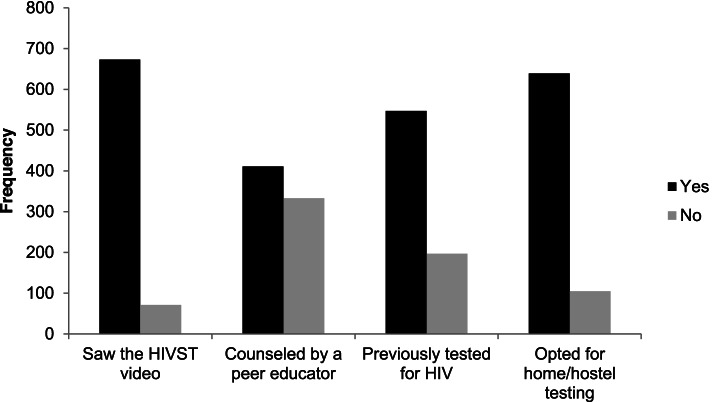
Characteristics of students who collected the HIVST kits

Figure 4 shows the frequencies of the characteristics of participants who collected an HIVST kit.

Using the chi-squared test, students who were aged below 20 years were less likely to have talked to a Peer Educator about HIVST before visiting the clinic (OR:0.6; 95% CI:0.4–0.8; *p* < 0.01) while those older than 24 years were more likely to opt for a hostel or home testing (OR:2.4 95% CI:1.5–3.9; *p* < 0.01).

**Fig. 5:**
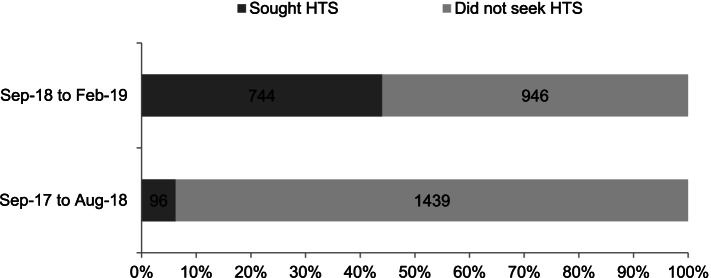
Frequency of students who sought HTS at the university clinic during the 12 months preceding the intervention versus the six-month intervention period

Figure 5 displays how the SAYS initiative increased the HTS uptake rate at the university clinic from 6.3% for the 12 months preceding the intervention to 44% for the 6 months of the intervention. It was difficult to ascertain the number of reactive results because none of the students who self-tested voluntarily disclosed their test results. 42% of the students who collected the HIVST kits gave inaccurate or non-existent mobile contacts. No harm was reported during the 6 months of HIVST kit disbursement.

While HIVST kits were only collected from the Peer Educators on duty at the clinic, participants also felt that the Peer Educators could be given kits to disburse beyond working hours as a way of accommodating those students with busy academic schedules. A Peer Educator explained: “Several students would visit my room after hours asking if I had extra kits because they had could not visit the clinic due to their cramped lecture timetables during the day. However, I always informed them about the lunchtime and weekend options.”

The HIVST program was also perceived as an opportunity for professional growth and engagement with adults and young adults. The following observation from one Peer Educator counselor was typical of their experience during the kit disbursement process: “Overall, leading the program awareness and counseling provided a platform for perfecting our counseling and leadership skills. I think this model proved that it is possible to create community peer network groups to spearhead and discuss issues that affect young people. These groups are better organized and supervised by professional counselors like health care workers.”

### Phase 3: summative evaluation survey

Data were collected from 349 conveniently sampled participants (who had or had not self-tested) and most of the students who participated in the survey were females (55%), and second years (51%). Of the students that participated in the summative evaluation survey, about 74% had self-tested during the 6 months of the SAYS Initiative. Approximately 97, 2, and 1% of the study participants reported their test results as negative, positive, and unknown, respectively. 74% of the participants either agreed or strongly agreed that HIVST kits should be made available to the general public. Approximately 57% had received an email on HIVST, 63% had read a pamphlet on HIVST, 66% had discussed HIVST with a Peer Educator, and 96% would encourage someone to self-test for HIV. For those who had tested for HIV during the SAYS Initiative, the major reasons for self-testing were convenience (56%) and privacy (40%) while those who did not self-test cited a lack of time (35%) and lack of interest (29%) as reasons for not self-testing. Of those who tested, most students (72%) cited that HIVST was easy to use. Nearly a quarter (23%) of those who did not test during the program said they did not think that they had the virus. To improve the uptake of HIVST survey participants suggested the need for more information on the self-testing program and ensuring the constant availability of test kits.

### Sustainment

About 88% of the summative evaluation survey participants who had self-tested reported that knowing their HIV status triggered positive changes to their participation in risky behavior and the majority (92%) recommended that the health facilities can safely offer free self-testing kits to all persons older than 18 years. The in-depth interviews were done 6 months post-intervention period revealed that the health providers, end-users, and institutions were eager to continue implementing the intervention.

When the SAYS Initiative terminated, there were limited funds to ensure consistency in the availability of HIVST kits. As a stop-gap measure, the university clinic partnered with an NGO which was supplying them with 50 HIVST kits monthly, though these were inadequate considering the increased demand for self-testing among the students. Within 3 months post-implementation, the institution managed to secure a stable partnership with the Global Health Global Ministries for another cycle of funding for training Peer Educators and purchasing more kits. There were concerns that the HIVST uptake could decline if the students were asked to buy the kits. To sustain the intervention in the long term, the implementers had to identify a reliable source of funding to ensure continued operational effectiveness.

The program findings were disseminated to the MoHCC for possible scaling up. The 24-h helpline was still active for students who needed post-test counseling services. Plans were put in place to organize quarterly sensitization workshops for the program implementers to influence longstanding change and reduce end-user recidivism.

## Discussion

This study describes the development and implementation of a coordinated multifaceted and multidisciplinary approach to improve the uptake of HTS among young people by integrating peer networking and professionals’ participation. Although traditional HTS have emphasized the importance of individuals’ knowing their HIV status, most of the population-specific testing has generally focused on children and mothers while missing other at-risk populations, like adolescents and young adults [30]. The study demonstrated the high demand, acceptability, and feasibility of an HIVST program among adolescents and young adults.

Despite studies showing the benefits of awareness of one’s HIV status, the prospect of stigma and negative mental sequel poses enormous implications on one’s decision to self-test. There is a need to disseminate messages assuring HIVST kit users about the resources available to support and educate them [31]. In this study, trained and dedicated program implementers provided tailored information on the intervention to dispel the barrier roots and enable end-users to give informed consent. Such information included offering the option of testing at the clinic, mutual support from trained Peer Educators, provision of a 24-h helpline for post-test services, and a follow-up text message. However, some students gave false or inaccurate phone contacts, an indirect indication that they were against the follow-up services which they possibly assumed would reduce confidentiality and anonymity. Similar reports were also noted in another study on HIVST conducted in Zimbabwe [32]. Although students were requested to anonymously use the helpline for post-test services, mHealth platforms may not be suitable for a small and geographically isolated communities like Africa University as students may not use the channel fearing diminished privacy, stigma, and gossip. Similar barriers of fear of identification and discomfort in receiving an HIV test from a clinician that the participant knew were also observed in two US studies [29, 33]. For HIV post-test services in small and isolated communities, we suggest the utilization of an off-site health facility to maintain confidentiality.

A study by Raquel Ramos and colleagues [34] recommended the involvement of peer groups from the study population in all aspects of design and implementation. The involvement of peers in the study was considered acceptable, practical, and essential by the students who participated in the study. The use of social network-based testing approach was also noted to be effective in a similar study that used friends and partners in promoting HIVST [19] and peer-to-peer HIV self-tests kit distribution by a trusted individual within the participants’ social network [35, 36]. Using peers can also decongest the health clinics particularly in low resource settings [19]. In addition, engaging Peer Educators during the planning and training processes also led to the standardization of the activities and ensured harmonization of communication messages across the implementation cascade. Tailored approaches such as contextualized HIVST pamphlets and the use of multimedia messaging enhances facilitate understanding of the HIVST process and also promote behavioural change [34, 37].

Understanding end users’ needs and preferences is critical in guiding effective HIVST interventions [38]. When planning HIVST programs among young people, implementers should be attentive to the packaging and instructions for the HIVST kits to be custom-made to the youths’ needs and local literacy levels and context [39, 40]. Packaging multilingual and youth-friendly (with graphic designs, colors, and minimal text) testing kits was an important factor in this study because the participants were from different language backgrounds and understanding testing instructions was a critical confidence booster during testing. Misunderstanding the instructions can lead to errors in interpreting test results [41, 42].

Although there was a comparable significant difference in uptake between the provider-delivered HIV testing statistics and HIVST, there were concerns over the proposed phone post-test counseling and linkage to care. This was also noted in Nigeria [40] and has been a major factor causing delayed adoption and expansion of HIVST programs in low-resource settings [43, 44]. Without the post-test service use, it was very difficult to monitor the use of kits and offer support services to those who tested. However, we noted that HIV testing programs often emphasized the clinical urgency of testing, as well as, target achievement while paying little attention to contextual and individual factors [45]. We recommend that HIVST delivery program implementers aim for context-specific and individualized strategies to encourage individuals to test and seek post-test services thereby avoiding inadvertent coercive testing associated with non-facility HIVST testing models [46]. This can provide a supportive and trusted environment for a broader tester buy-in among suboptimal testers.

Increasing the number of adolescents and young adults who know their HIV status can be accelerated by coordinated and organized strategies that are feasible, acceptable, and sustainable to them. The efficacy of deliberately organized and advertised HIVST programs has previously been proved to yield positive results in youths [19, 47, 48]. Using trained kit distributers like Peer Educators in program implementation offered individual support and reduced clinical service demands and allowed clinicians to focus their expertise on complex case management [49]. Disconnecting testing from clinical facility settings and reducing interactions with health providers was predicted to diminish negative perceptions associated with HIV testing experiences [31].

Effective HIVST interventions require subsidization or external funding to sustain population access and coverage [50]. The sustainability of the program requires additional research studies and a consistent supply of kits. This study was conducted over a short interval and the noted difference between the facility-based and peer-delivered HIVST can motivate enthusiasm for a broader randomized multi-site implementation of the HIVST program for profound comparisons. Young and unemployed adults have limited access to finances and attaching a price to the kits can potentially deter the students from self-testing [47]. Costing the HIVST kits should be given thorough consideration during intervention development if the uptake of HTS among adolescents and young adults is to be improved [40]. Besides, HIVST delivering organizations can create long term partnerships with organizations promoting HIVST to sustain free or low-cost kit distribution.

The findings of this study can assist institutions of higher learning and similar settings which are planning to implement or scale up the HIVST program. Implementers will easily follow through the SAYS Initiative phases, identify gaps and possible solutions before the actual program implementation. A peer network-based strategy will be crucial to achieving the UNAIDS 95–95-95 targets, particularly the first 95.

### Strengths and limitations

The study used several data collection methods and this enabled data triangulation and increased the likelihood that the perspectives of the different sources reflected practical realities of the intervention in this study setting. Furthermore, the study was co-designed and delivered by peers who collaborated with a multidisciplinary team of researchers, clinicians and young people living with HIV. The blended partnerships were key to understanding facilitators of HTS uptake among adolescents and young adults. It is worth mentioning that this study had several limitations. The intervention was implemented at one private institution of higher learning and the study population may not be representative of university students in Zimbabwe. Thus, the small sample sizes for the different phases may limit the breadth of the findings. Using self-reporting for data collection during the surveys could have also resulted in recall bias and socially desirable responses. The poor uptake of post-test services by students failed to capture participants’ experiences of self-testing and we were not able to track the students who opted for off-site post-testing services. However, the use of ‘special bins’ to dispose of the used kits at the hostels provided an estimate of the use of the kits. Nevertheless, the findings of this study were consistent with other studies in similar contexts [51–54].

## Conclusion

Peer delivered HIVST using trained personnel was acceptable among adolescents and young adults offered the intervention at a campus setting. A high degree of HIVST acceptability among young people is a strong advantage and drive towards increased access through scaling up the intervention. The present study demonstrated that HIVST could increase the uptake of HIV testing for this population given the stigma associated with facility-based HTS and the need for routine HIV testing for this age group who may be reticent and may not otherwise test. Youth-friendly packaging, free distribution, and easy accessibility of the HIVST kits have a major bearing in reaching young people at a small and geographically isolated community like a university campus. An off-site post-test counseling option is likely to improve the implementation of a campus-based HIVST and close the linkage to treatment and care gap.

## Supplementary Information

**Additional file 1.** HIV self testing project: training of trainers facilitators manual hand book.

**Additional file 2.** Training Evaluation Form.

## Data Availability

The datasets generated or analyzed during the current study are available from the corresponding author on reasonable request.

## Abbreviations

CATS: Community adolescents treatment support
EBP: Evidence-based practice
HIV/AIDS: Human Immuno-deficiency Virus/ Acquired Immuno-deficiency Syndrome
HIVST: HIV Self-testing
HTS: HIV testing services
MoHCC: Ministry of Health and Child Care, Zimbabwe
NGO: Non-governmental organization
SAYS: Swipe And Know Your Status
WHO: World Health Organization

## References

[1] Joint United Nations Programme on HIV/AIDS. The gap. Report. 2014. https://files.unaids.org/en/media/unaids/contentassets/documents/unaidspublication/2014/UNAIDS_Gap_report_en.pdf. Accessed 9 Aug 2021.

[2] McHugh G, Koris AL, Bandason T, Kranzer K, Ferrand RA (2020). Uptake of HIV self-testing amongst youth in tertiary education colleges in Zimbabwe. Top Antivir Med.

[3] Nkomazana N, Maharaj P (2014). Perception of risk of HIV infections and sexual behaviour of the sexually active university students in Zimbabwe. SAHARA-J J Soc Asp HIV/AIDS.

[4] Emeka-Nwabunnia I, Ibeh BO, Ogbulie TE (2014). High HIV sero-prevalence among students of institutions of higher education in Southeast Nigeria. Asian Pacific J Trop Dis.

[5] World Health Organization. Adolescent health. Geneva: WHO; 2021. https://www.who.int/health-topics/adolescent-health#tab=tab_1. Accessed 19 Aug 2021.

[6] Zimbabwe National Statistics Agency and ICF international. Zimbabwe demographic and health survey 2015: final report. Zimbabwe National Statistics Agency (ZIMSTAT) and ICF International. Rockville; 2016.

[7] Fleming PJ, Rosen JG, Wong VJ, Carrasco MA (2019). Shedding light on a HIV blind spot: factors associated with men’s HIV testing in five African countries. Glob Public Health.

[8] Chung AH, Rimal RN (2015). Applying choice architecture principles to understand HIV testing: findings from Malawi and Zimbabwe. AIDS Educ Prev.

[9] Gazimbi MM, Magadi MA (2019). Individual- and community-level determinants of antenatal hiv testing in Zimbabwe. J Biosoc Sci.

[10] Maman S, van Rooyen H, Stankard P, Chingono A, Muravha T, Ntogwisangu J (2014). NIMH project accept (HPTN 043): results from in-depth interviews with a longitudinal cohort of community members. PLoS One.

[11] Alonzo A, Reynolds NR (1995). Stigma, HIV and AIDS: an exploration and elaboration of a stigma trajectory. Soc Sci Med.

[12] Tafuma TA, Mahachi N, Dziwa C, Moga T, Baloyi P, Muyambo G (2018). Barriers to HIV service utilisation by people living with HIV in two provinces of Zimbabwe: results from 2016 baseline assessment. South Afr J HIV Med.

[13] Gombe N (2018). Barriers to HIV testing among adolescents and young adults in Harare City. Zimbabwe Texila Int J Public Heal.

[14] Choko AT, MacPherson P, Webb EL, Willey BA, Feasy H, Sambakunsi R (2015). Uptake, accuracy, safety, and linkage into care over two years of promoting annual self-testing for HIV in Blantyre, Malawi: a community-based prospective study. PLoS Med.

[15] Choko AT, Desmond N, Webb EL, Chavula K, Napierala-Mavedzenge S, Gaydos CA (2011). The uptake and accuracy of Oral kits for HIV self-testing in high HIV prevalence setting: a cross-sectional feasibility study in Blantyre, Malawi. PLoS Med.

[16] Wong V, Johnson C, Cowan E, Rosenthal M, Peeling R, Miralle M (2014). HIV self-testing in resource-limited settings: regulatory and policy considerations. AIDS Behav.

[17] World Health Organization. Guidelines on HIV self-testing and partner notification: Supplement to Consolidated Guidelines on HIV Testing Services. 2016. https://www.ncbi.nlm.nih.gov/books/NBK401684/. Accessed 20 Aug 2021. 27977094

[18] HIVST.org. A clearing house of information on HIV self-testing to further collaboration between global public health stakeholders. HIV Self-testing Research and Policy Hub. 2017. http://hivst.org/. Accessed 20 Aug 2021.

[19] Pettifor A, Lippman SA, Kimaru L, Haber N, Mayakayaka Z, Selin A (2020). HIV self-testing among young women in rural South Africa: a randomized controlled trial comparing clinic-based HIV testing to the choice of either clinic testing or HIV self-testing with secondary distribution to peers and partners. EClinicalMedicine..

[20] Hatzold K, Mutseta M, Sibanda E, Gudukeya S, Tumushime M, Lopez C, et al. Closing the HIV testing gap: Facility-based integration of HIV self-testing, a way to improve testing coverage, yield and efficiency of client-initiated HIV testing services in Zimbabwe. UNITAID-PSI. 2017;6. https://www.psi.org/wp-content/uploads/2020/06/POSTER-Hatzold-et-al-Facility-based-integration-HIVST-Zimbabwe.pdf. Accessed 19 Aug 2021.

[21] Njau B, Covin C, Lisasi E, Damian D, Mushi D, Boulle A (2019). A systematic review of qualitative evidence on factors enabling and deterring uptake of HIV self-testing in Africa. BMC Public Health.

[22] Cowan F, Ncube G, Mugurungi O, Hatzold K, Mavengere Y, Sibanda E, et al. Supervised HIV self-testing to inform implementation and scale up of self-testing in Zimbabwe. J Int AIDS Soc. 2015;18(4)

[23] World Health Organization. Market and technology landscape: HIV rapid diagnostic tests for self-testing. Vernier; 2017. http://unitaid.org/assets/HIV-Rapid-Diagnostic-Tests-for-Self-Testing_Landscape-Report_3rd-edition_July-2017.pdf. Accessed 13 July 2021.

[24] Leask CF, Sandlund M, Skelton DA, Altenburg TM, Cardon G, Chinapaw MJM (2019). Framework, principles and recommendations for utilising participatory methodologies in the co-creation and evaluation of public health interventions. Res Involv Engagem.

[25] Green LW, O’Neill M, Westphal M, Morisky D (1996). The challenges of participatory action research for health promotion. Promot Educ.

[26] Craig P, Dieppe P, Macintyre S, Michie S, Nazareth I, Petticrew M (2013). Developing and evaluating complex interventions: the new Medical Research Council guidance. Int J Nurs Stud.

[27] Ministry of Health and Child Care (2017). Operational and service delivery manual for the prevention, care and treatment of HIV in Zimbabwe.

[28] Aarons GA, Hurlburt M, Horwitz SM (2011). Advancing a conceptual model of evidence-based practice implementation in public service sectors. Adm Policy Ment Heal Ment Heal Serv Res.

[29] Lippman SA, Moran L, Sevelius J, Castillo LS, Ventura A, Treves-kagan S (2017). Acceptability and feasibility of HIV self-testing among transgender women in San Francisco: a mixed methods pilot study. AIDS Behav.

[30] Kellerman SE, Ahmed S, Feeley-Summerl T, Jay J, Kim M, Phelps BR (2013). Beyond prevention of mother-to-child transmission: keeping HIV-exposed and HIV-positive children healthy and alive. AIDS..

[31] Mathews A, Farley S, Conserve DF, Knight K, Le’marus A, Blumberg M (2020). “Meet people where they are”: a qualitative study of community barriers and facilitators to HIV testing and HIV self-testing among African Americans in urban and rural areas in North Carolina. BMC Public Health.

[32] Dziva Chikwari C, Bernays S, Dringus S, Simms V, Weiss HA, Sibanda E (2020). Addressing the challenges and relational aspects of index-linked HIV testing for children and adolescents: insights from the B-GAP study in Zimbabwe. Implement Sci Commun.

[33] Marks SJ, Merchant RC, Clark MA, Liu T, Rosenberger JG, Bauermeister JA, et al. Barriers to HIV testing and opportunities for expansion using home-based HIV self-Testing : results of a National Study of higher HIV risk young men who have sex with men. SAGE Open. 2021;11(2)

[34] Raquel Ramos S, Lardier DT, Bond KT, Boyd DT, O’hare OM, Nelson LE, et al. Participatory design of a web-based hiv oral self-testing infographic experiment (Hotie) for emerging adult sexual minority men of color: a mixed methods randomized control trial. Int J Environ Res Public Health 2021;18(22). 10.3390/ijerph182211881 PMC8618392 34831644

[35] Iwelunmor J, Ezechi O, Obiezu-Umeh C, Gbaja-Biamila T, Nwaozuru U, Oladele D (2020). The 4 youth by youth HIV self-testing crowdsourcing contest: a qualitative evaluation. PLoS One.

[36] Matovu JKB, Nambuusi A, Nakabirye S, Wanyenze RK, Serwadda D (2020). Formative research to inform the development of a peer-led HIV self-testing intervention to improve HIV testing uptake and linkage to HIV care among adolescents, young people and adult men in Kasensero fishing community, Rakai, Uganda: a qualitative study. BMC Public Health.

[37] Bond KT, Ramos SR. Utilization of an animated electronic health video to increase knowledge of post- and pre-exposure prophylaxis for HIV among African American women: Nationwide cross-sectional survey. JMIR Form Res. 2019;3(2) 10.2196/formative.9995 PMC6658301 31144667

[38] Mcguire M, De WA, Karellis A, Janssen R, Engel N, Sampath R (2021). EClinicalMedicine HIV self-testing with digital supports as the new paradigm : A systematic review of global evidence ( 2010–2021 ). EClinicalMedicine..

[39] Simwinga M, Kumwenda MK, Dacombe RJ, Kayira L, Muzumara A, Johnson CC, et al. Ability to understand and correctly follow HIV self-test kit instructions for use: applying the cognitive interview technique in Malawi and Zambia. J Int AIDS Soc. 2019;22(1) 10.1002/jia2.25253 PMC6432102 30907496

[40] Obiezu-Umeh C, Gbajabiamila T, Ezechi O, Nwaozuru U, Ong JJ, Idigbe I (2021). Young people’s preferences for HIV self-testing services in Nigeria: a qualitative analysis. BMC Public Health.

[41] Wei C, Yan L, Li J, Su X, Lippman S, Yan H (2018). Which user errors matter during HIV self-testing? A qualitative participant observation study of men who have sex with men (MSM) in China. BMC Public Heal.

[42] Nash SG, Maffeo M, Likatavicius G, Cosmaro L, Rudaitis K, Lapsinov A (2021). Acceptability and usability of HIV self-tests in two European countries : findings from surveys of clients at non-governmental organisations in Lithuania and Italy. BMC Infect Dis.

[43] Choko AT, Jamil MS, MacPherson P, Corbett E, Chitembo L, Ingold H (2020). Measuring linkage to HIV treatment services following HIV self-testing in low-income settings. J Int AIDS Soc.

[44] Ekouevi DK, Bitty-Anderson AM, Gbeasor-Komlanvi FA, Coffie AP, Eholie SP (2020). HIV self-testing: the key to unlock the first 90 in west and Central Africa. Int J Infect Dis.

[45] UNAIDS. Ending AIDS: progress towards the 90–90–90 targets. Joint U Nations Program HIV/AIDS (UNAIDS). 2017. https://www.unaids.org/en/resources/documents/2017/20170720_Global_AIDS_update_2017. Accessed 19 Aug 2021.

[46] Ong JJ, Li H, Dan W, Fu H, Liu E, Ma W (2018). Coercion and HIV self-testing in men who have sex with men: implementation data from a cross-sectional survey in China. J Acquir Immune Defic Syndr.

[47] Indravudh PP, Sibanda EL, D’elbée M, Kumwenda MK, Ringwald B, Maringwa G (2017). “I will choose when to test, where I want to test”: investigating young people’s preferences for HIV self-testing in Malawi and Zimbabwe. AIDS..

[48] Hecht J, Sanchez T, Sullivan PS, Dinenno EA, Cramer N, Delaney KP (2021). Increasing access to HIV testing through direct-to-consumer HIV self-test. Morbility Mortal Wkly Rep.

[49] Williams OD, Dean JA, Crothers A, Gilks CF, Gow J (2021). Economic evaluation of alternative testing regimes and settings to detect undiagnosed HIV in Australia. BMC Health Serv Res.

[50] Bell SFE, Lemoire J, Debattista J, Redmond AM, Driver G, Durkin I (2021). Online HIV self-testing (HIVST) dissemination by an australian community peer HIV organisation: a scalable way to increase access to testing, particularly for suboptimal testers. Int J Environ Res Public Health.

[51] Mokgatle MM, Madiba S (2017). High acceptability of HIV self-testing among technical vocational education and training college students in Gauteng and north West Province: what are the implications for the scale up in South Africa?. PLoS One.

[52] Tonen-Wolyec S, Mbopi-Kéou F-X, Batina-Agasa S, Kalla GCM, Noubom M, Bouassa R-SM, et al. Acceptability of HIV self-testing in African students: a cross-sectional survey in the Democratic Republic of Congo. PAMJ. 2019;33(83) 10.11604/pamj.2019.33.83.18586 PMC6689849 31448045

[53] Koris AL, Stewart KA, Ritchwood TD, Mususa D, Ncube G, Ferrand RA (2021). Youth-friendly HIV self-testing: acceptability of campus-based oral HIV self-testing among young adult students in Zimbabwe. PLoS One.

[54] Zhou B, Bentham J, Di CM, Bixby H, Danaei G, Cowan MJ (2017). Worldwide trends in blood pressure from 1975 to 2015: a pooled analysis of 1479 population-based measurement studies with 19·1 million participants. Lancet..

