# Supplementary material for: Implementation of a campus-based and peer-delivered HIV self-testing intervention to improve the uptake of HIV testing services among university students in Zimbabwe: the SAYS initiative
Source: BMC Health Serv Res. 2022 Feb 18;22:222. doi: 10.1186/s12913-022-07622-1 (PMC8855554; doi:10.1186/s12913-022-07622-1)
Supplement: Supplementary file 1 — Additional file 1. HIV self testing project: training of trainers facilitators manual hand book. [file 12913_2022_7622_MOESM1_ESM.docx]

**HIV SELF TESTING PROJECT: TRAINING OF TRAINERS FACILITATORS MANUAL HAND BOOK**

**OBJECTIVES**

- To equip AU Peer Educators Trainers with further facilitation and life skills.
- To deepen participants’ knowledge and understanding of the Behavior Change, HIV and HIV Self Testing (HIVST) (BC-HIVST) so that they can facilitate the project awareness among their peers.
- To provide an opportunity for interaction and sharing of experiences with other peers.
- To provide an opportunity for the facilitators to deepen their own spiritual life and renew their commitment to help create an AIDS free generation and to promote HIVST among youths and young adults

**What is facilitation?**

- It is helping individuals/participants to discover things for themselves.
- Bringing out what is already within the participants.
- Empowering participants to make their own decisions
- Enabling

**HOW?**

By:

- Beginning with what the participants already know and believe in.
- Moving onto a critical analysis of practical experiences.
- Introducing new information and ideas on HIVST and HCT.
- Assisting participants to make a responsible and informed choice.

**Facilitation is NOT**

- Giving information only.
- Imposing your own personal views.
- Preaching to people.
- Having all the answers.

**Some of good qualities of a counselor**

- Alive Exemplary
- Humble Observant
- Respectful Good listener
- Patient Audible and clear
- Prayerful Prepares well & psychological well
- Dependable Honest
- Punctual

**Some DON’TS in facilitation**

- Arguing
- Interrupting or talking too much
- Pass judgement
- Give advice, unless requested for.
- Ignore the speaker
- Filter what is said
- Rehearse your response
- Sarcasm
- Let the speaker’s emotions react too directly on your own.
- Express shock.

**DOs in facilitation**

- Give your full attention
- Try to hear all that is said-i.e. tone of voice and body language.
- Show real interest and true desire.
- Keep a kind and smiling expression.
- Be understanding.
- Listen for the cause of the problem.
- Help the speaker associate the problem with the cause.
- Occasionally ask hard questions.
- Cultivate the ability to be silent when silence is needed.

**Effective facilitation should**

- Create a good learning environment.
- Initiate discussion and arouse interest.
- Guide the discussion through suggesting new directions.
- Lead the group.
- Provide factual information.
- Summarize
- Evaluate

**Qualities of a good facilitator**

**A good facilitator should:**

- be well prepared whilst remaining flexible
- think and act creatively
- deal with sensitive issues and manage people’s feelings
- encourage humor and respect
- negotiate with and influence others
- keep to time without being driven by it
- A facilitator is like a conductor.
- Great music emerges when everyone is communicating together
- Good communication, just like good music, is more about listening than talking.
- Facilitation is the art, not of putting ideas into people’s heads, but of drawing ideas out.

**NOTE THAT!**

**THE ROLE OF THE FACILITATOR REMAINS ALWAYS THAT OF HELPING THE PARTICIPANTS TO FIND PERSONAL SATISFYING ANSWERS. THE DISCUSSION JUST PROVIDES INFORMATION AND DEEPENED UNDERSTANDING.**

**COMMUNICATION**

- The two-way movement of information, ideas or feelings in such a way that both parties perceive its identical meaning.

**Barriers to communication**

- Noise Stereotyping
- Culture Dressing
- Education Accent
- Lack of confidence Lack of knowledge
- Sex and gender Language
- Lack of trust Talking too much
- Being aggressive Anger / emotions
- Embarrassment

**Essential skills for a facilitator**

**Questioning**

- It exposes assumptions
- Brings out false beliefs
- Manifests generalizations
- Seeks clarifications to unsubstantiated opinions
- Brings out the truth
- A good facilitator listens closely and responds with open-ended eliciting questions in order to explore what would have been said e.g.
- Aren’t you assuming that everybody understand the risks associated with sex?
- Are you saying then that tradition is now irrelevant to society?
- If what you are saying is correct, wouldn’t it follow that life is now unpredictable?

**Paraphrasing**

Repeating in your own words, what you understand to have been said, in order to assure the speaker that they have been understood. e.g.

- In other words you are saying that…
- So, you are implying that….

**Encouraging**

- The facilitator’s use of verbal and non-verbal signals to create a climate for discussion and trust building.
- This involves the nodding of the head, maintaining eye contact and open body posture.

**Summarizing**

- Periodically noting key points, ideas and feelings.
- Winding up the discussion.
- Establishing a basis for further discussion.
- Reviewing progress.
- Checking for clarity and agreement.

**THE L.E.A.D. MODEL**

- Leaders can use a four step model to ensure participation of all members.

**L**- lead with a clear purpose

**E**- empower to participate

**A**- aim for consensus

**D**- direct the process

**Key leadership functions of the model**

- Setting clear goals and objectives.
- Getting people involved.
- It assures that there is power within the group to make decisions.
- Participation and consensus also help maintain individual self esteem and encourage open communication.
- Reaching consensus on important items which helps building of mutual trust and achieve a healthy respect for differences among team members
- Empowering members to participate achieves the high level of interaction and involvement that group members need.
- Provides a platform for constructive conflict resolution

**Positive Reinforcement**

- Be specific about what you are praising.
- Be timely; do not wait too long after the event.
- Keep praise separate from problems or negative concerns, it may get lost if it is sandwiched between problems.
- Give praise regularly but not so often that it becomes expected or meaningless

**How to use videos as a teaching aid**

- Have clear objectives in the use of each video.
- Set the context and organize the follow-up discussion so that participants can criticize, analyze and share information contained in the video.

**Key steps in showing video**

- Preview the tape to familiarize self with the video content e.g. the HIVST video.
- Ensure that the television or projector is placed in a position where each person can hear and see clearly.
- Explore what the participants already know about the topic before showing the video.
- Give a brief overview of the video content.
- Encourage participants to listen carefully.
- Strategically position yourself where you can observe viewers’ reactions as this will help you in the discussion.

**After the show**

1. Ask the viewers for their overall reaction to the show. e.g.
2. What are your feelings, and reactions towards the video?
3. Was the film true to life?
4. Write up the responses on the newsprint.
5. Discuss the new information gained from the video.
6. Summarize and conclude.

**ORAQUICK: HIV SELF-TEST**

**What is OraQuick HIV Self-Test?**

1. The OraQuick HIV Self-Test (HIVST) is a rapid, point-of-care test that allows an individual to detect antibodies to both HIV -1 and HIV -2 with a simple oral swab.
2. WHO definition of HIVST: HIV Self-Testing is a process in which a person collects his/her own specimen (oral fluid) and then performs a test and interprets the result, often in private setting either alone or with someone he/she trusts. (WHO, HIV SELF-TESTING and partner Notification, Guidelines on HIV Testing Services (2016:14).
3. Provides a result in as little as 20 minutes in the privacy of an individual at health centers.
4. Oral test does not provide final HIV diagnosis, anyone who tests HIV positive using HIVST must undergo another different test to confirm diagnosis prior to being treated for HIV. (WHO 2016 guidelines on HIVST).
5. OraQuick HIVST is not recommended for people on ART as there is a chance that someone may test negative and stop taking their medication. This is because people on medication can achieve viral suppression where the virus becomes undetectable, but remains latent in the body.

**Why HIVST is being offered now?**

1. HIVST is offered as an additional approach to HIV testing services.
2. HIVST will contribute to closing the testing gap and achieving the UN 95-95-95 and 2030 global goals.
3. HIVST may provide people especially the young adults with additional pathways to HIV prevention, care and treatment. HIVST also shares many characteristics with current HTC approaches including linkages to prevention and care services.
4. HIVST provides confidentiality and empowers users to be responsible for their own HIV status.

**WHO 5 Cs of HIV Testing services will apply to HIVST**

1. **Consent:**  All people receiving HIVST kits must give their informed consent to take up the test and be counseled
2. **Confidentiality:** HIVST should be confidential. Any discussion between health provider or peer educator and the student should not be disclosed to a third party without the express consent of person being tested.
3. **Counseling :** Pre-test information and post test counseling can be provided at individual level or in a group setting if appropriate, however all persons should have an opportunity to ask questions in a private setting if they request it.
4. **Correct test results:** Provider of HTS should strive to provide high-quality testing services with QA mechanisms to ensure people receive correct diagnosis.
5. **Connection:** Linkage to prevention, treatment and care services should include the provision of effective and appropriate follow-up.

NOTE: The actual process of HIVST, step by step will be done after protocol approval by MRCZ. This is a general training of trainers peer educators information.
