# Supplementary material for: Implementation of a campus-based and peer-delivered HIV self-testing intervention to improve the uptake of HIV testing services among university students in Zimbabwe: the SAYS initiative
Source: BMC Health Serv Res. 2022 Feb 18;22:222. doi: 10.1186/s12913-022-07622-1 (PMC8855554; doi:10.1186/s12913-022-07622-1)
Supplement: Supplementary file 2 — Additional file 2. Training Evaluation Form. [file 12913_2022_7622_MOESM2_ESM.doc]

# Date: ____________

**Training Evaluation Form**

For *participants* in HIVST Training

# Title and location of training: _____________________________________________________________________________ Participant Name-Optional: __________________________________________________

**Instructions:** Please indicate your level of agreement with the statements listed below in #1‐11.

Strongly Agree

Agree Neutral Disagree

Strongly Disagree

1. The objectives of the training were clearly defined.

    

1. Participation and interaction were encouraged.
2. The topics covered were relevant to me.
3. The content was organized and easy to follow.

    

    

    

1. The materials distributed were helpful.
2. This training experience will be useful in my work.
3. The trainer was knowledgeable about the training topics.
4. The trainer was well prepared.

    

    

    

    

1. The training objectives were met.
2. The time allotted for the training was sufficient.
3. The meeting room and facilities were adequate and comfortable.

    

    

    

1. What did you like most about this training?
2. What aspects of the training could be improved?
3. How do you hope to change your practice as a result of this training?
4. What additional adult ESL trainings would you like to have in the future?
5. Please share other comments or expand on previous responses here:

# Thank you for your feedback!
